# Supplementary material for: The Integrated Effect of Environmental Conditions and Human Presence on the Behaviour of a Pair of Zoo-Housed Asian Small-Clawed Otters
Source: Animals (Basel). 2023 Jul 6;13(13):2228. doi: 10.3390/ani13132228 (PMC10340003; doi:10.3390/ani13132228)
Supplement: Supplementary file 1 [file animals-13-02228-s001.zip › animals-2437970-supplementary.pdf]

Table S1. Full results of Generalized Linear Mixed Models on the effects of environmental/individual factors and human presence on the otters' behavioral categories (N_{cases}=168).
Note: $p < 0.05$ is boldface.

**Predictors**	**Estimates**	**SEM**	χ^2	**p**
		**GLMM₂ (*vigilance*)**		
Intercept[a]	-7.981	3.191	a	a
Animal identity1[b]	0.083	0.149	0.555	0.579
Session 2[b]	-1.698	0.349	-4.867	**<0.001**
Session 3[b]	-2.479	0.391	-6.333	**<0.001**
Session 4[b]	-1.719	0.375	-4.578	**<0.001**
Session 5[b]	-1.603	0.356	-4.498	**<0.001**
Session 6[b]	-1.683	0.312	-5.393	**<0.001**
Staff presence	0.234	0.343	0.681	0.496
THI	0.056	0.029	1.965	**0.050**
Noise	0.049	0.046	1.071	0.284
Visitor score	0.730	1.061	0.688	0.492
Noise * Visitor score	-0.009	0.017	-0.565	0.572
		**$GLMM_4$ (*out of sight$_{indoor}$*)**		
Intercept[a]	-0.361	4.427	a	a
Animal identity1[b]	-0.178	0.196	-0.909	0.364
Session 2[b]	1.033	0.462	2.237	**0.025**
Session 3[b]	2.139	0.501	4.273	**<0.001**
Session 4[b]	0.453	0.484	0.935	0.350
Session 5[b]	0.846	0.464	1.824	0.068
Session 6[b]	1.018	0.409	2.491	**0.013**
Staff presence	-0.115	0.487	-0.236	0.814
THI	-0.052	0.039	-1.340	0.180
Noise	0.057	0.069	0.829	0.407
Visitor score	0.792	1.791	0.442	0.658
Noise * Visitor score	-0.016	0.028	-0.596	0.551
		**$GLMM_5$ (*out od sight$_{outdoor}$*)**		
Intercept[a]	-5.118	3.250	a	a
Animal identity1[b]	0.394	0.126	3.116	**0.002**
Session 2[b]	-0.588	0.313	-1.881	0.060
Session 3[b]	-1.698	0.370	-4.590	**<0.001**
Session 4[b]	-0.031	0.347	-0.090	0.928

Session 5[b]	-0.460	0.329	-1.397	0.162
Session 6[b]	-0.819	0.280	-2.922	**0.003**
Staff presence	0.459	0.285	1.608	0.108
THI	0.049	0.031	1.565	0.118
Noise	-0.014	0.045	-0.322	0.747
Visitor score	-0.781	1.035	-0.755	0.450
Noise * Visitor score	0.015	0.016	0.907	0.365
**GLMM$_6$ (*locomotion*)**				
Intercept[a]	-4.771	5.637	a	a
Animal identity1[b]	0.075	0.113	0.664	0.507
Session 2[b]	-0.567	0.289	-1.964	**0.005**
Session 3[b]	-1.325	0.363	-3.650	**<0.001**
Session 4[b]	-0.278	0.404	-0.687	0.492
Session 5[b]	-0.306	0.316	-0.967	0.333
Session 6[b]	-0.475	0.290	-1.635	0.102
Staff presence	0.576	0.344	1.674	0.094
THI	0.031	0.026	1.203	0.229
Noise	-0.012	0.091	-0.129	0.898
Visitor score	-0.050	2.262	-0.022	0.982
Noise * Visitor score	0.002	0.035	0.061	0.952
**GLMM$_9$ (*caregiver-otter interaction*)**				
Intercept[a]	-5.785	2.581	a	a
Animal identity1[b]	0.077	0.115	0.672	0.502
Session 2[b]	-0.791	0.253	-3.129	**0.002**
Session 3[b]	-0.432	0.278	-1.555	0.120
Session 4[b]	-0.136	0.280	-0.486	0.627
Session 5[b]	-0.381	0.250	-1.521	0.128
Session 6[b]	-0.461	0.216	-2.134	**0.033**
Staff presence	1.467	0.249	5.880	**<0.001**
THI	0.009	0.023	0.396	0.692
Noise	0.001	0.036	0.037	0.971
Visitor score	0.307	0.840	0.362	0.717
Noise * Visitor score	-0.004	0.013	-0.275	0.783
**GLMM$_{10}$ (*visitor-otter interaction*)**				
Intercept[a]	-5.486	3.063	a	a
Animal identity1[b]	-0.240	0.106	-2.249	**0.002**
Session 2[b]	-0.191	0.269	-0.710	0.478

Session 3[b]	-0.473	0.305	-1.549	0.121
Session 4[b]	0.027	0.299	0.091	0.927
Session 5[b]	0.262	0.272	0.960	0.337
Session 6[b]	0.088	0.243	0.360	0.719
Staff presence	0.122	0.292	0.419	0.675
THI	0.027	0.031	0.865	0.387
Noise	-0.028	0.052	-0.547	0.585
Visitor score	-0.966	1.643	-0.588	0.557
Noise * Visitor score	0.018	0.025	0.729	0.466
**$GLMM_{11}$ (*juggling*)**				
Intercept[a]	-5.341	3.214	a	a
Animal identity1[b]	0.801	0.149	3.569	**<0.001**
Session 2[b]	-0.409	0.336	-1.218	0.223
Session 3[b]	-0.621	0.350	-1.776	0.076
Session 4[b]	0.074	0.341	0.218	0.828
Session 5[b]	-0.108	0.331	-0.327	0.743
Session 6[b]	-0.445	0.295	-1.507	0.132
Staff presence	-0.109	0.327	-0.333	0.739
THI	0.041	0.028	1.464	0.143
Noise	-0.025	0.045	-0.566	0.571
Visitor score	-0.420	1.059	-0.397	0.691
Noise * Visitor score	0.009	0.016	0.540	0.589
**$GLMM_{12}$ (*ARBs*)**				
Intercept[a]	-5.146	3.191	a	a
Animal identity1[b]	-0.030	0.139	-0.212	0.832
Session 2[b]	0.549	0.313	1.752	0.080
Session 3[b]	0.005	0.350	0.014	0.990
Session 4[b]	-0.098	0.356	-0.274	0.784
Session 5[b]	-0.128	0.335	-0.384	0.701
Session 6[b]	-0.121	0.290	-0.416	0.677
Staff presence	0.888	0.294	3.025	**0.002**
THI	0.017	0.028	0.614	0.539
Noise	-0.010	0.045	-0.230	0.818
Visitor score	-0.010	1.076	-0.009	0.993
Noise * Visitor score	0.001	0.016	0.050	0.960

[a]Not shown as not having a meaningful interpretation.
[b]These predictors were dummy-coded, with the reference category as follow: Animal identity = 0; Session = Session 1.
